# Graft Diameter Should Reflect the Size of the Native Anterior Cruciate Ligament (ACL) to Improve the Outcome of ACL Reconstruction: A Finite Element Analysis

**DOI:** 10.3390/bioengineering9100507

**Published:** 2022-09-27

**Authors:** Huizhi Wang, Mingzhu Tao, Qinyi Shi, Kaixin He, Cheng-Kung Cheng

**Affiliations:** School of Biomedical Engineering and Engineering Research Center for Digital Medicine of the Ministry of Education, Shanghai Jiao Tong University, Shanghai 200030, China

**Keywords:** anterior cruciate ligament reconstruction (ACLR), graft diameter, graft force, knee stability

## Abstract

The size of the anterior cruciate ligament (ACL) often varies between individuals, but such variation is not typically considered during ACL reconstruction (ACLR). This study aimed to explore how the size of the ACL affects the selection of a suitable graft diameter. A finite element model of a human knee was implanted with intact ACLs of different dimensions (0.95, 1 and 1.05 times the size of the original ACL) and with grafts of different diameters, to simulate ACLR (diameter 7.5–12 mm in 0.5 mm increments). The knee models were flexed to 30° and loaded with an anterior tibial load of 103 N, internal tibial moment of 7.5 Nm, and valgus tibial moment of 6.9 Nm. Knee kinematics (anterior tibial translation (ATT), internal tibial rotation (ITR) and valgus tibial rotation (VTR)) and ligament forces were recorded and compared among the different groups. The results showed that, compared with the intact knee, a graft diameter of 7.5 mm was found to increase the ATT and VTR, but reduce the graft force. Increasing the graft diameter reduced knee laxity and increased the graft force. A 10% increase in the size of the ACL corresponded to a 3 mm larger graft diameter required to restore knee stability and graft force after ACLR. It was concluded that the graft diameter should be selected according to the dimensions of the native ACL, for better restoration of knee functionality. This study may help to improve the clinical treatment of ACL ruptures.

## 1. Introduction

The anterior cruciate ligament (ACL) is a critical stabilizer for the knee, especially for constraining anterior tibial translation (ATT) and internal tibial rotation (ITR) [1,2,3]. However, the ACL is prone to injury during high-intensity sports, such as skiing, basketball and football [4]. ACL reconstruction (ACLR) is a surgical treatment that replaces the torn ACL with a graft, with the aim of restoring normal knee kinematics and function. However, the diameter of commonly used ACL autografts, such as the popular four-strand hamstring tendon, are often determined by tissue volume obtained from the donor site, without considering the suitability of this graft diameter for replacing the function of the native ACL [5]. From a biomechanical perspective, an unsuitable graft diameter may not restore the normal function of the native ACL and thus may induce abnormal knee kinematics and excessive force on the graft. This may, further, lead to post-operative complications, such as graft re-rupture and long-term knee osteoarthritis (OA) derived from abnormal articular stress, which are both frequently reported complications after ACLR [6,7].

Previous studies have reported that larger graft diameters may improve the outcomes of ACLR. Snaebjornsson et al. reported that each incremental increase of 0.5 mm in graft diameter resulted in an 86% lower likelihood of needing revision surgery [8]. Wang et al. reported that larger graft diameters can be beneficial for restoring joint stability and graft force and for reducing stress concentrations on the articular cartilage [9]. Park et al. showed that a graft diameter of 8.0 mm or larger can result in a relatively better outcome of ACLR in terms of knee functional scores and rate of revision surgery [10].

However, Fujimaki et al. reported a large variation in the dimensions of the human ACL [11], which indicated that the graft diameter may need to be selected individually to adequately restore knee function. However, few studies have explored how individual variation in the size of the native ACL affects the selection of graft diameter. In addition, studies on the potential correlation between ACL and auto-grafts are scarce. Pujol et al. reported that the effective diameters of auto-hamstring tendons obtained during surgery are approximately 10% larger than the ACL isthmus [12]. Even if assuming the size of an auto-hamstring graft is proportional to the size of the ACL, it is still unclear whether it is adequate for restoring knee function.

This study aimed to explore how the size of the native ACL affects the selection of graft diameter, chosen with the aim of restoring knee stability and graft force. It was hypothesized that a 10% increase in the size of the native ACL would require a considerably larger graft diameter to restore knee stability and graft force. The outcome of this study may elucidate whether or not it is important to choose the graft diameter according to the individual size of the native ACL. This may provide a scientific basis for improving surgical techniques for treating ACL ruptures and improving clinical outcomes.

## 2. Materials and Methods

A finite element model of a human cadaveric knee was simulated with different-sized native ACLs (95%, 100% and 105% of the size of the native ACL) and after ACLR using hamstring tendon grafts with different diameters (7.5–12 mm in 0.5 mm increments). The same loading condition was applied to each model, after which knee kinematics and ligament forces were compared among the groups, to explore the differences in suitable graft diameter for replacing the different-sized ACL.

### 2.1. Finite Element Model of the Intact Knee

**Figure 1:**
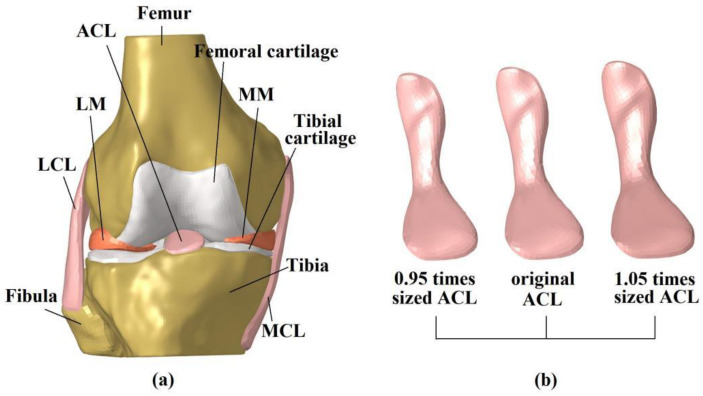
3D model of the intact knee (**a**), ACL models showing different sizes (**b**). LM: lateral meniscus; MM: medial meniscus; LCL: lateral collateral ligament; MCL: medial collateral ligament.

A previously developed and validated model of a human cadaveric knee (male, 45 years, right leg) was used in this study [13]. The three-dimensional (3D) geometry of the joint tissues was reconstructed from MRI images (scan resolution of 0.2 × 0.2 mm with a slice thickness of 0.2 mm, field of view measuring 12 cm × 8 cm × 10 cm, FLASH pulse sequence, field strength of 3.0 T, and with TE = 26.3 ms and TR = 53 ms), using Mimics 10.01 (Materialise N.V., Leuven, Belgium). The model included the femur, tibia, fibula, articular cartilage, menisci, ACL, posterior cruciate ligament (PCL), medial collateral ligament (MCL) and lateral collateral ligament (LCL) (Figure 1a). The model was meshed in HyperMesh 12.0 (Altair Engineering, Tokyo, Japan) using 4-node tetrahedron elements. Mesh convergence testing verified that an element size of 1 mm was small enough to eliminate the effect of element size on the accuracy of model calculation. The final model had a total number of 659,251 elements. Bones (Young’s modulus = 400 MPa, and Poisson’s ratio v = 0.33) and cartilage (Young’s modulus = 5 MPa, and Poisson’s ratio v = 0.46) were assumed to be linear isotropic elastic. Menisci were assumed to be orthotropic elastic tissues (E_θ_ = 125 MPa, E_R_ = E_Z_ = 27.5 MPa, G_θR_ = G_θZ_ = 2 MPa, G_RZ_ = 10.34, V_θR_ = V_θZ_ = 0.1 and V_RZ_ = 0.33). Ligaments were defined as isotropic hyperelastic, using strain energy functions. The Veronda-Westmann function was used for the ACL and PCL (α_ACL_ = 0.3 MPa, β_ACL_ = 12.20, α_PCL_ = 0.18 MPa and β_PCL_ = 17.35), while the Mooney–Rivlin material model was used to define the mechanical properties of the MCL and LCL. The coefficients for the LCL were assumed to be identical to those of the MCL (C1 = 30.1 MPa and C2 = −27.1 MPa). The calculation accuracy of the model was validated using biomechanical data from a cadaveric experiment on the same knee sample. The model was able to calculate the ATT, valgus tibial rotation (VTR) and in situ force in the ACL with an accuracy of 0.1 mm, 1° and 1 N, respectively [13].

### 2.2. Simulation of Intact Knee Using Different-Sized ACLs

To simulate different sizes of the ACL, the original geometry of the cadaveric ACL was proportionally shrunk, and enlarged, by 5% (Figure 1b), using the software Creo Parametric 7.0 (Parametric Technology Corporation, Boston, MA, USA). The contact surfaces of the ACL and insertion point in the bones (femur and tibia) were connected by a “Tie” constraint, which ensured there was no relative displacement or rotation between the connected elements. The effective diameter and cross-sectional area of the bone insertion sites and isthmus of each ACL model are displayed in Table 1. The effective diameter (D) was defined as follows:

(1) $$D=2\times \sqrt{(\frac{S}{\pi })}$$

where S represents the cross-sectional area of the bone insertion sites or the isthmus of the ACL.

### 2.3. Simulation of ACLR Using Different Graft Diameters

**Figure 2:**
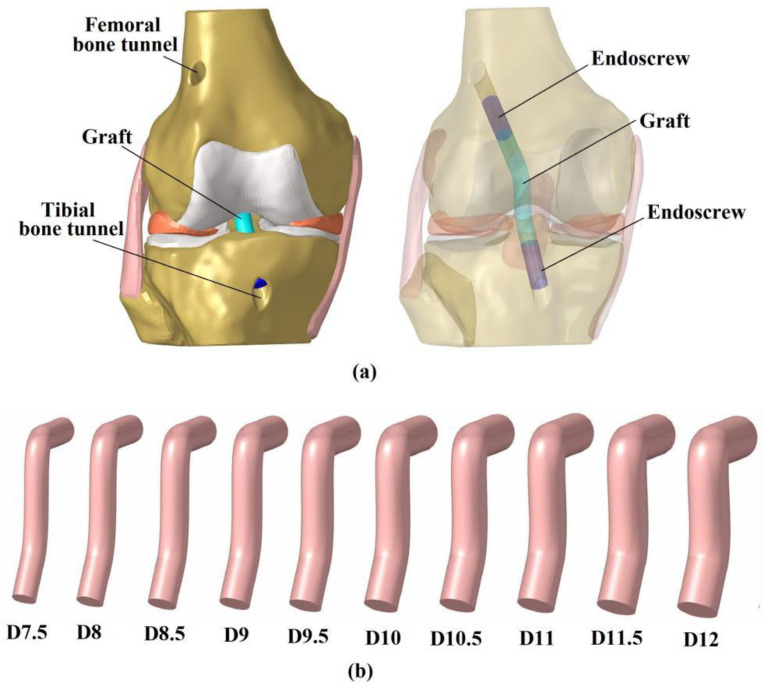
The 3D model of the knee after ACL reconstruction (**a**). Graft models with different diameters (**b**). D7.5 represents a graft diameter of 7.5 mm.

This study simulated anatomical single-bundle ACLR, using different graft diameters (Figure 2). The centers of the femoral- and tibial bone insertion sites of the native ACL were set as the entrances for the bone tunnels. The angles between the femoral tunnel axis and the horizontal and sagittal planes were 45° and 25°, respectively, and the angles between the tibial tunnel axis and the horizontal and sagittal planes were 65° and 25°, respectively. The grafts were modeled as cylindrical, to simulate the geometry of an auto- hamstring tendon graft and secured in the bone tunnel with an Endoscrew of length 10 mm and with the same diameter as the graft [14]. One end of the Endoscrew was tied with the end of the graft and the exterior wall of the screw was tied with the bone tunnel wall to simulate a secure fixation. The length of the graft inside the femoral tunnel was 20 mm and inside the tibial tunnel was 10 mm, due to the shorter tunnel. The Endoscrew had a Young’s modulus of 110 GPa and Poison’s ratio of 0.35, to simulate a titanium material.

Given that the diameter of hamstring tendon grafts are approximately 10% larger than the ACL isthmus [12], the resulting graft for ACLR may have an effective diameter of 7.5 mm (with a 6.7 mm diameter at the ACL isthmus cross-section). In this study, the diameter of the hamstring tendon graft was defined as a variable, ranging from 7.5 to 12 mm, increasing in increments of 0.5 mm (Figure 2). The grafts were assigned a Young’s modulus of 168 MPa and a Poisson’s ratio of 0.45 [15].

### 2.4. Boundary and Loading Conditions and Simulation Outputs

**Figure 3:**
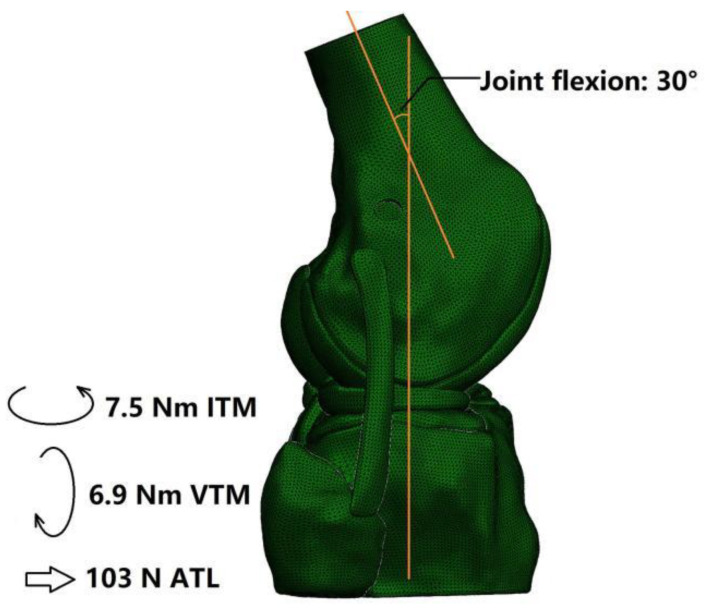
Boundary and loading conditions. ITM: internal tibial moment; VTM: valgus tibial moment; ATL: anterior tibial load.

The simulations were conducted using finite element analysis in Abaqus/CAE 6.14-2 (Simulia, Inc., Waltham, MA, USA) and all models were subject to the same boundary and loading conditions (Figure 3). First, the bottom surface of the tibial shaft was fixed in 6 degrees of freedom (DOFs) and the femur was flexed to 30°. Next, the femoral shaft was fixed in 6 DOFs while the tibial shaft was fixed only in flexion–extension. The tibial shaft was then subjected to an anterior tibial load of 103 N, internal tibial moment of 7.5 Nm and valgus tibial moment of 6.9 Nm. These loads represent the maximum values experienced during normal gait (15% body weight for ATL, 1.1% body weight for ITM and 1% body weight for VTM), as a worst case for tensioning the ACL during walking, where its function was best examined [16].

Joint kinematics under the above loading conditions were recorded to evaluate knee stability, including ATT, ITR and VTR. Greater knee displacement than the intact state indicated knee instability in the corresponding DOFs, which may cause an abnormal stress distribution on the articular cartilage, and consequently induce knee OA in the long-term [9]. The in situ forces in the ACL and grafts were also obtained, to evaluate the restoration of normal functionality. A lower force borne by the graft, in comparison to the native ACL, would indicate insufficient restoration, which may produce greater loading in other joint tissues and increase the risk of secondary damage.

## 3. Results

### 3.1. ACLR vs. Intact Knee with Original-Sized ACL

Knee kinematics and ligament forces from the intact knee and the knee after ACLR are shown in Table 2. Compared with the intact knee, ACLR using a graft diameter of 7.5 mm resulted in greater ATT (4.4 mm vs. 4.2 mm) and VTR (1.3° vs. 0.9°), but a lower in situ force in the graft (115 N vs. 128 N). ITR was restored to a near-physiological level (11.8° vs. 12.1°). Increasing the graft diameter resulted in a reduction in ATT, ITR and VTR, and led to greater force on the graft. ATT, VTR and graft force were restored to physiological levels when using graft diameters of 9 mm, 12 mm and 10.5 mm, respectively.

### 3.2. Outcome for the Intact Knee Groups with Different Sized ACL

**Figure 4:**
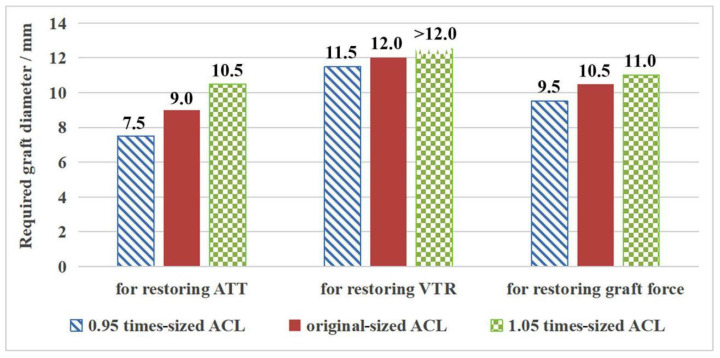
Graft diameters required to restore knee kinematics and graft force, for knees with different-sized native ACL.

As shown in Table 3, a larger ACL (1.05 times the original size) resulted in a reduction in ATT, ITR and VTR (2%, 2% and 11%, respectively) and a greater force on the ACL (5%). In contrast, a smaller ACL resulted in greater laxity of the joint and a lower ACL force. Correspondingly, the graft diameter required to restore knee stability and graft force increased as the size of the ACL increased. Knee stability, in terms of internal tibial rotation (ITR), could be restored by using a graft diameter of 7.5 mm. Figure 4 shows the required graft diameter for restoring the ATT, VTR and graft force for the different sized ACL groups. It was found that in order to restore ATT to a physiological level, the knee required a graft diameter of 7.5 mm, 9.0 mm and 10.5 mm, respectively, when using an ACL sized at 0.95, 1 and 1.05 times that of the native ACL. Similarly, graft diameters of 11.5 mm and 12.0 mm were required to restore VTR to within range of the intact knee with an ACL 0.95 and 1 times that of the native ACL. However, even the largest graft diameter of 12.0 mm could not achieve a VTR value similar to the knee with a 1.05 times-sized ACL. The graft force could be restored to normal levels by using graft diameters of 9.5 mm, 10.5 mm and 11.0 mm for the three groups (0.95-, 1-, 1.05 times-sized ACL), respectively.

In general, larger graft diameters were required to restore VTR, while restoring ITR required the smallest graft diameters. Compared with the original-sized ACL group, the required graft diameter for restoring knee stability and graft force in the 0.95 times-sized ACL group decreased by 0.5–1.5 mm, and that for the 1.05 times-sized ACL group increased by 0.5–1.5 mm (Figure 4). Compared with the 0.95 times-sized ACL group, the 1.05 times-sized ACL group needed a 1.5–3 mm larger graft diameter to restore knee stability and graft force.

Table 4 shows the graft diameter required to restore the graft force and knee stability (ATT, ITR and VTR) to a physiological level. By comparing the size of the graft and native ACL, it was found that the required graft diameter was generally 0.5 mm larger than the average effective diameter of the bone insertion sites and isthmus of the native ACL.

## 4. Discussion

This study found that knees with a large ACL, similarly, required a large diameter graft to restore knee stability and graft force after ACLR. A 10% difference in the size of the ACL resulted in a difference of 1.5–3 mm in the required graft diameter. Considering such a big difference in required graft diameter and a rather narrow range in graft diameters used clinically (usually 6–11 mm [17]), this study suggests that grafts should be chosen according to the size of the native ACL, to reduce the risk of postoperative complications such as knee instability, graft re-rupture and long-term OA. The results also indicate that the average diameter of the femoral and tibial insertion sites, and the isthmus of the ACL, might be good indicators of a suitable graft diameter. The outcomes of this study need to be further verified by conducting cadaveric and in vivo studies in a larger population sample, before transitioning to clinical practice. 

Recent studies have indicated that the “one-size-fits-all” principle may not be suitable for patients whose physiology is in the tails of the bell curve of human anatomy [18]. However, few studies have quantitatively evaluated the importance of matching graft diameter to the size of the native ACL, as presented in this report. This study showed that ACLR using a common graft diameter (7.5 mm for the individual model used in this study) resulted in a greater ATT and VTR and a lower graft force than the intact knee. The greater movement (ATT and VTR) indicates knee instability in these DOFs, which in the long term may cause knee OA. Barenius et al. [7] reported that the incidence of knee OA after ACLR increased three-fold in comparison to the contralateral healthy knee at 14 years after surgery. In addition to the lack of constraint of the knee joint, the lower force in the graft may also cause excessive loading on the other joint tissues. Increasing the graft diameter was found to improve knee stability and increase the graft force, which may reduce post-operative complications and improve the clinical outcome. These results are supported by Snaebjornsson et al. [8]. In general, a larger diameter may allow more load to transfer through the graft.

The results of this study showed a relatively large variation in the required graft diameter when used to replace native ACLs of different sizes. Studies have shown a large individual variation in the dimensions of the native ACL, with the CSA at the isthmus ranging from 35.1 to 103.6 mm^2^ [19], and ranges of 113–156 mm^2^ and 111–176 mm^2^ for the femoral and tibial insertion sites, respectively [11,20,21,22]. According to our findings, the large variation in the size of the ACL indicates that there should be a correspondingly large variation in the required graft diameter. By comparing the graft diameter and dimensions of the native ACL, this study found that the required graft diameter was generally 0.5 mm larger than the average effective diameter of the bone insertion sites and isthmus of the native ACL, indicating that the latter might be a good indicator for choosing a suitable graft diameter. However, previous studies have also shown that cartilage damage was reduced when using stiffer grafts [23], which means that both the diameter and mechanical properties can heavily influence the outcomes of ACLR, and the graft properties may impact the selection of a suitable graft diameter. Therefore, defining quantitative criteria for choosing a suitable graft (according to the size of the native ACL) needs further consideration. What is more, it is difficult to measure the dimensions of the native ACL in a clinical setting, mostly because of the limited resolution of current medical imaging techniques. However, recent studies [24] have found a significant correlation between the dimensions of the ACL and several bony structures in the knee joint, indicating a promising method of inferring ACL dimensions by measuring the bone parameters on a single X-ray image. In the context of the above findings, it is suggested that the graft properties are quantified before use, rather than just its diameter. The criteria for choosing a suitable graft diameter should also be tested accordingly, for clinical applications. This might be challenging when using autografts, since it is difficult to run mechanical tests on the graft prior to use. Therefore, allografts and artificial grafts might be more promising for quantitative evidence-based ligament reconstruction and for ensuring a better clinical outcome. In terms of artificial grafts produced from novel biomaterials, similar computational simulations and further clinical validation studies should be conducted before applying such novel products to clinical use. 

It should be noted that when VTR was restored to a physiological level, both ATT and ITR were over-restrained and the graft force was greater than the value recorded for the intact ACL. Over-restraining of ATT and ITR may present as a stiffness in the knee in these DOFs, which may hinder daily activities that require good flexibility of the knee joint. A larger force in the graft may make it vulnerable to rupture, which would require revision surgery. These concerns warrant further study into improving ACLR techniques, thus reducing the risk of asynchronous restoration of knee kinematics and graft force. This study also showed that even the large graft diameter of 12 mm could not restore VTR in line with the 1.05 times-sized ACL. A 12 mm graft diameter was the largest size in this study because expanding the tibial bone tunnel any further would have damaged the tibial cartilage.

This study has some limitations: (1) The smaller and larger sizes of the ACL were modeled by proportionally scaling the ACL geometry without changing the morphology of other joint tissues. In reality, the morphology of the other joint structures, especially the contour of the articular surfaces, may also show individual variation, which might affect knee kinematics and function. However, the method used in this study ensured that the size of the ACL was the only variable from which conclusions were drawn. This is one of the advantages of using finite element analysis over cadaveric or in vivo studies. The strict control of the variables allows for individual differences to be excluded and negates any impact on the characteristic being studied. Therefore, statistical analysis was not considered necessary for this study. (2) All grafts were considered to have isotropic material properties. This does not accurately reflect all graft materials, particularly those from tissues commonly displaying anisotropic properties. Nevertheless, the main function of the ACL is to bear tensile forces and the conditions used in this study adequately simulated physiological loading, which primarily acted to tension the ACL and grafts. Assigning isotropic properties to the ligaments is not expected to affect the main finding of this study. (3) The internal tibial rotation (ITR) was possibly over-constrained in this FE model, in comparison to the native ACL, because the longitudinal fibers of the ligament were not simulated, and thus the rotation of fibers could not be simulated. This may result in a lower simulated ITR. (4) The finite element model was simplified in comparison to the complex human joint, and the model geometry was adopted from a single sample. However, finite element analysis allows the variate to be changed an infinite number of times, without damaging the sample, and the variate can be strictly controlled to one factor when the other factors are kept constant. This permits the basic joint biomechanics to be examined more clearly. 

## 5. Conclusions

This study shows that a minor difference in the size of the ACL can have a considerable effect on the graft diameter required to restore knee stability. Thus, it is necessary to choose the graft diameter according to the size of the native ACL, with the averaged diameter—calculated from the ACL insertion sites and the isthmus—being a good indicator of a suitable graft diameter. The findings of this study may help to improve the clinical treatment of ACL ruptures.

## Figures and Tables

**Table 1:** Effective diameter (D) and cross-sectional area (S) of the bone insertion sites and the isthmus of the ACL models.

	Femoral Insertion		Isthmus		Tibial Insertion	
	D (mm)	S (mm^2^)	D (mm)	S (mm^2^)	D (mm)	S (mm^2^)
0.95 times sized ACL	11.8	109.5	6.4	31.9	14.6	167.5
Original ACL	12.4	121.3	6.7	35.3	15.4	185.6
1.05 times sized ACL	13.0	133.7	7.0	38.9	16.2	204.6

**Table 2:** Anterior tibial translation (ATT), internal tibial rotation (ITR), valgus tibial rotation (VTR) and ACL/graft force in the intact knee with a native-sized ACL and in the knee after anterior cruciate ligament reconstruction (ACLR) using different graft diameters.

		ATT (mm)	ITR (°)	VTR (°)	ACL/Graft Force (N)
Native ACL		4.2	12.1	0.9	128
Diameter of the graft in ACLR (mm)	7.5	4.4	11.8	1.3	115
	8	4.3	11.6	1.2	116
	8.5	4.3	11.5	1.2	120
	9	4.2	11.4	1.2	122
	9.5	4.2	11.3	1.2	124
	10	4.2	11.2	1.1	127
	10.5	4.1	11.0	1.1	129
	11	4.1	10.9	1.1	134
	11.5	4.0	10.7	1.0	139
	12	3.8	10.4	0.9	147

**Table 3:** Anterior tibial translation (ATT), internal tibial rotation (ITR), valgus tibial rotation (VTR) and ACL/graft force in the intact knee with different sized ACL.

	ATT (mm)	ITR (°)	VTR (°)	ACL/Graft Force (N)
0.95 times-sized ACL	4.4	12.1	1.0	124
Native ACL	4.2	12.1	0.9	128
1.05 times-sized ACL	4.1	11.9	0.8	134

**Table 4:** The graft diameter ($D_{\mathrm{stability}}$, in mm) required to restore graft force and knee stability in anterior tibial translation (ATT), internal tibial rotation (ITR) and valgus tibial rotation (VTR), and the average values of effective diameters ($\frac{D_{F}+D_{\mathrm{Is}}+D_{T}}{3},\;$ in mm) of the femoral and tibial insertion sites and isthmus of the native ACL.

	$D_{stability}\;(\mathrm{mm})$	$\frac{D_{F}+D_{Is}+D_{T}}{3}\;(\mathrm{mm})$
0.95 times-sized ACL	11.5	10.9
Original-sized ACL	12.0	11.5
1.05 times-sized ACL	>12.0	12.1

## Data Availability

All data have been included in the manuscript.
